# Using the Drug Burden Index to identify older adults at highest risk for medication-related falls

**DOI:** 10.1186/s12877-020-01598-5

**Published:** 2020-06-12

**Authors:** Susan J. Blalock, Chelsea P. Renfro, Jessica M. Robinson, Joel F. Farley, Jan Busby-Whitehead, Stefanie P. Ferreri

**Affiliations:** 1 Division of Pharmaceutical Outcomes and Policy, UNC Eshelman School of Pharmacy, University of North Carolina at Chapel Hill, Chapel Hill, NC CB# 7573 USA; 2 Department of Clinical Pharmacy and Translational Science, University of Tennessee Health Science Center College of Pharmacy, Memphis, USA; 3 Division of Practice Advancement and Clinical Education, UNC Eshelman School of Pharmacy, University of North Carolina at Chapel Hill, Chapel Hill, USA; 4 Department of Pharmaceutical Care & Health Systems, University of Minnesota College of Pharmacy, Minneapolis, USA; 5 Division of Geriatric Medicine and Director, Center of Aging and Health, UNC School of Medicine, University of North Carolina at Chapel Hill, Chapel Hill, USA

**Keywords:** Medication therapy management, Accidental falls, Aging, Health services, Medication

## Abstract

**Background:**

The Drug Burden Index (DBI) was developed to assess patient exposure to medications associated with an increased risk of falling. The objective of this study was to examine the association between the DBI and medication-related fall risk.

**Methods:**

The study used a retrospective cohort design, with a 1-year observation period. Participants (*n* = 1562) were identified from 31 community pharmacies. We examined the association between DBI scores and four outcomes. Our primary outcome, which was limited to participants who received a medication review, indexed whether the review resulted in at least one medication-related recommendation (e.g., discontinue medication) being communicated to the participant’s health care provider. Secondary outcomes indexed whether participants in the full sample: (1) screened positive for fall risk, (2) reported 1+ falls in the past year, and (3) reported 1+ injurious falls in the past year. All outcome variables were dichotomous (yes/no).

**Results:**

Among those who received a medication review (*n* = 387), the percentage of patients receiving at least one medication-related recommendation ranged from 10.2% among those with DBI scores of 0 compared to 60.2% among those with DBI scores ≥1.0 (Chi-square (4)=42.4, *p* < 0.0001). Among those screened for fall risk (*n* = 1058), DBI scores were higher among those who screened positive compared to those who did not (Means = 0.98 (SD = 1.00) versus 0.59 (SD = 0.74), respectively, *p* < 0.0001).

**Conclusion:**

Our findings suggest that the DBI is a useful tool that could be used to improve future research and practice by focusing limited resources on those individuals at greatest risk of *medication-related* falls.

## Background

Falls are the leading cause of injury-related morbidity and mortality among older adults worldwide [1, 2]. Past research demonstrates that polypharmacy (i.e., use of multiple medications) increases the risk of falling [3, 4]. Typically, studies designate individuals taking 4+ or 5+ medications as the high-risk group [5]. Currently, no consensus definition of polypharmacy exists, however [6]. Beyond the sheer number of medications used, many specific medications have been associated with an increased risk of falling [7–10]. These are commonly referred to as fall-risk-increasing-drugs (FRIDs) [11–13]. Recent systematic reviews and meta-analyses suggest that the evidence for increased fall risk is strongest and most consistent for sedatives and hypnotics, antidepressants, and benzodiazepines [9, 10]. There is also consistent evidence that cumulative exposure to anticholinergics increases the risk of falling [11]. Cumulative exposure is assessed by scales that weight each medication a patient is taking by dosage or strength of anticholinergic activity and sum the resulting values across all the medications the patient is taking with anticholinergic properties [11, 14]. Other classes of medications (e.g., cardiovascular agents, antidiabetics, nonsteroidal anti-inflammatories) have been implicated as risk factors for falling and they are often included in indices designed to capture exposure to FRIDS despite the lack of consistent evidence that they actually increase risk [15].

Multifactorial fall prevention programs, which have been shown to reduce both the risk and rate of falls, typically include a component focused on medication review and modification [16]. The Stopping Elderly Accidents, Deaths, & Injuries (STEADI) Initiative developed by CDC recommends that all individuals who screen positive for fall risk receive a medication review and that medications likely to increase fall risk be optimized by discontinuing the medication, switching to a lower risk medication, or reducing dosage of the medication to the lowest effective level [17]. Only three published studies have examined the effectiveness of community-based, single component fall prevention interventions focused on medication review and modification [18–20]. None of these studies demonstrated a statistically significant difference in the rate or risk of falls between individuals in the control and intervention groups. Two of the studies had a sample size less than 200 [18, 19]. Therefore, lack of power may have contributed to the null findings reported [21].

Statistical power is affected by many factors in addition to sample size, including the rate at which the primary outcome occurs in the control group. When the event rate is low, more participants are needed to achieve the same level of power to detect a between-group difference of a specified size [22]. This is particularly relevant for the design of fall prevention interventions focused on medication review and modification. Falls can be caused by many factors including both intrinsic (e.g., vision deficits, lower extremity weakness, cognitive impairment, incontinence), and extrinsic (e.g., medications, footwear, environmental factors such as floor rugs, poor lighting, and tripping hazards) [23]. Interventions focused on medication review and modification, however, target a single factor – medication use. Thus, the event rate of interest is not simply rate of falls, but the rate of medication-related falls. Potentially, power in intervention studies could be improved by using inclusion/exclusion criteria that maximize this rate. This is challenging, however, due to the lack of consensus on the specific medications that pose the greatest risk. Notably, the three previous studies that evaluated the effect of fall prevention interventions focused on medication review and modification used different eligibility criteria: a fall within the previous 12 months without regard to medication use [19]; use of 4+ medications including at least one with central nervous system activity [18]; and use of at least one FRID, where FRIDs included cardiovascular agents and other medications where there is a lack of consistent evidence that they actually increase fall risk.

In the study reported in this paper, we examine whether the Drug Burden Index (DBI) might provide a tool to identify individuals at increased risk for medication-related falls in future intervention studies. The DBI is a validated measure used to assess a person’s total exposure to medications with anticholinergic and sedative properties, including most medications that have been associated with an increased risk of falling [24]. In past research, the DBI has been shown to predict falls and other functional outcomes among older adults [25–35]. Potentially, the DBI could be used in clinical settings to identify older adults at high risk for medication-related falls [36].

## Methods

### Design

We used a retrospective cohort study design, with a 1-year look-back observation period. Data were derived from a randomized controlled trial designed to evaluate whether a community pharmacy-based medication management intervention targeting adults age 65 and older reduced (1) the use of medications associated with an increased risk of falling and (2) fall-related emergency room and urgent care visits. (This trial was nested within a larger effort sponsored by the Centers for Medicare and Medicaid Innovation to organize and encourage community pharmacies to provide enhanced services to Medicaid and Medicare recipients in North Carolina). A total of 65 community pharmacies participated in the trial; 34 were randomized to the control group and 31 to the intervention group. Patient prescription records were used to identify individuals who were: (1) age 65 or older, (2) filled at least 80% of their prescriptions at a participating pharmacy, and (3) used either four or more chronic medications or at least one medication associated with an increased risk of falling. A total of 10,565 patients were identified.

For this paper, we limited the sample to older adults who received their medications from one of the pharmacies assigned to the intervention group (*n* = 4719) and used data only from the 1-year period prior to intervention implementation. In addition, because medication use was assessed using Medicare Part D and North Carolina (NC) Medicaid claim records, we limited the sample to intervention group participants who had continuous coverage for prescription medications through either Medicare Part D or NC Medicaid for the entire 1-year period of observation (*n* = 1562).

Each intervention pharmacy received a spreadsheet listing the names of patients served by the pharmacy who met study inclusion criteria. Pharmacy staff screened patients by asking the following key questions derived from the STEADI Initiative [17]: (1) Have you fallen in the past year, (2) Do you feel unsteady when standing or walking, and (3) Do you worry about falling. In addition, patients who reported one or more falls within the past year were asked if any of the falls had resulted in injury. Patients who answered “Yes” to any of the key STEADI questions were classified as having screened positive for increased fall risk. These patients were eligible to receive a medication review provided by a pharmacist associated with the pharmacy where they obtained their medications. As part of the medication review, the pharmacist evaluated the patient’s medication regimen using evidence-based algorithms developed by the study team to: (1) identify medications associated with an increased risk of falling and (2) provide therapeutic recommendations to reduce risk [37]. Pharmacists conducting the medication reviews were aware of participants’ responses to the key STEADI questions. After conducting a medication review, the pharmacist communicated recommendations to the patient’s prescriber using forms developed for this purpose. The study was approved by the Institutional Review Board at the University of North Carolina at Chapel Hill.

### Measures

We used the DBI to assess each participant’s cumulative exposure to medications with anticholinergic or sedative properties during the 1-year observation period using information from Medicare Part D and NC Medicaid claims records (i.e., medication name, strength, dosage form, date dispensed, quantity dispensed, days supply). For each claim record involving a medication with sedative or anticholinergic properties, we calculated a DBI score using the following formula, $$ DBI=\raisebox{1ex}{$\ D$}\!\left/ \!\raisebox{-1ex}{$\left(D+\delta \right)$}\right. $$, where D is the patient’s daily dose of the medication and *δ* is the minimum recommended daily dose for the medication for any indication approved by the Food and Drug Administration. Daily dosage was determined by the strength of the medication dispensed multiplied by the quantity dispensed and divided by the days supply indicated in the prescription claim record. For each month during the 1-year observation period, these values were summed across all of the medications the patient was taking with sedative or anticholinergic properties to yield a single patient-level score. We then summed these monthly values and divided the total by 12 to obtain an average monthly DBI score for each patient.

Our primary outcome indexed whether at least one medication-related recommendation (switch to a different medication, discontinue medication, reduce medication dose) was communicated to a participant’s health care provider following a medication review. We used this variable as an indicator of whether falls reported by study participants were potentially medication-related. Only participants who screened positive for fall risk were eligible to receive a medication review. The data used to classify participants on this outcome were derived from standardized forms completed by pharmacy staff. Participants were coded 1 if they: (a) received a medication review and (b) the review resulted in at least one medication-related recommendation being communicated to their health care provider. Participants were coded 0 on this variable if they: (a) received a medication review and (b) no medication-related recommendations were communicated to their health care provider following the review. Participants who did not have a medication review were coded as missing on this variable.

We also examined three secondary outcomes: whether the participant reported having fallen at least once during the past year, whether the participant screened positive for fall risk by responding *Yes* to at least one of three STEADI screening questions, and whether the participant reported experiencing any injurious falls in the past year. All responses were coded dichotomously (1 = Yes/0 = No). Finally, we assessed three control variables: patient age (in years), patient gender, and the average number of prescriptions filled each month during the 1-year observation period.

### Analyses

Characteristics of study participants are presented using means and percentages, depending on the measurement properties of the variables. We used both bivariate analyses (e.g., t-tests, chi-squared tests) and multivariable logistic regression models to assess the relationship between the DBI and outcome variables. In the logistic regression analyses, we ran a separate regression model for each outcome. Because DBI scores were positively skewed, we treated it as a categorical variable with 5 levels: 0 [reference group] (*n* = 245), Low: > 0 to < 0.20 (*n* = 244), Moderate: 0.20 to < 0.50 (*n* = 240), High: 0.50 to < 1.0 (*n* = 352), and Very High: ≥ 1.0 (*n* = 481). Each model also included age, gender, and average number of prescriptions filled/month as control variables. We ran parallel models with the control variables excluded to assess the potential impact of confounding. We also performed power analyses to explore the effect of potentially using the DBI in future studies to restrict the sample to individuals at greater risk for medication-related falls. In these analyses, we calculated the number of participants needed to achieve a power of 0.80 assuming different baseline levels of fall risk. All analyses were performed using the Statistical Analysis System for Personal Computers (SAS PC) version 9.4 (SAS Institute, Inc., Cary, NC).

## Results

Of the 1562 patients included in the sample, 1058 (67.7%) were screened for fall risk using the STEADI questions. Patients who were screened did not differ from those who were not screened with respect to: age (Mean (Standard Deviation, SD) = 74.9 (8.1) versus 75.6 (8.4), *p* = 0.14), average number of prescriptions filled/month (Mean (SD) = 6.4 (4.1) versus 6.2 (4.0), *p* = 0.26), or average monthly DBI score (Mean (SD) = 0.81 (0.92) versus 0.84 (0.89), *p* = 0.66), respectively.

### Bivariate analyses

**Fig. 1:**
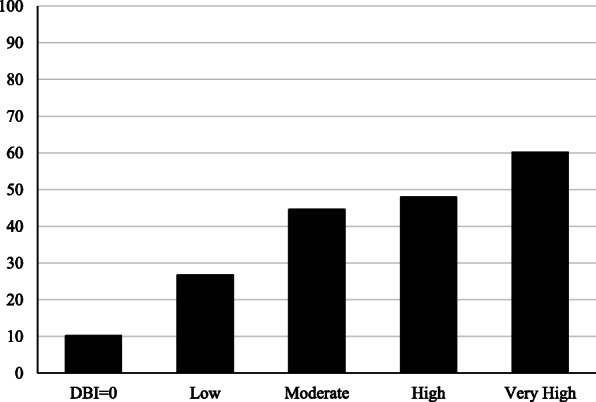
Probability of receiving at least one medication-related recommendation following the medication review (*n* = 387). Notes. DBI scores categorized as: Reference Group: 0, Low: > 0 to < 0.20, Moderate: 0.20 to < 0.50, High: 0.50 to < 1.0, and Very High: ≥ 1.0

Among patients who received a medication review (*n* = 387), DBI scores were nearly two times higher among those for whom the review resulted in at least one medication-related recommendation being communication to their health care provider compared to those where no medication-related recommendations were made. (Table 1) As shown in Fig. 1, the probability of receiving at least one medication-related recommendation varied from 10.2% among those with a DBI score of 0 to 60.2% among those with a DBI score ≥ 1.0 (Chi-square (4)=42.4, *p* < 0.0001).

**Table 1:** Bivariate relationships between Drug Burden Index and outcome variables

Variable^†^		n	%	DBI Score Mean^‡^ (SD)	DBI Score Median^‡^ (IQR)
1+ medication recommendations communicated to prescriber after medication review (*n* = 387)	Yes	171	44.2	1.20 (1.03)^*^	0.95 (1.18)^*^
	No	216	55.8	0.69 (0.82)	0.46 (0.91)
Screened Positive (n = 1058)	Yes	609	57.6	0.98 (1.00)*	0.66 (1.17)*
	No	449	42.4	0.59 (0.74)	0.32 (0.92)
1+ fall within the past year (*n* = 1057)	Yes	312	29.5	1.11 (1.05)*	0.87 (1.19)*
	No	745	70.5	0.69 (0.82)	0.46 (0.97)
1+ injurious fall within the past year (*n* = 1057)	Yes	170	16.1	1.17 (1.05)*	0.95 (1.20)*
	No	887	83.9	0.75 (0.87)	0.50 (1.02)

^†^ For the medication recommendation variable, n is limited to patients who received a medication review. N varies across screening questions due to missing data

^‡^Differences involving means and medians were evaluated using independent group t-tests and Kruskal-Wallis tests, respectively. *SD* Standard Deviation, *IQR* Interquartile range

^*^P < 0.0001

Among all patients screened (*n* = 1058), DBI scores were higher among patients who (1) screened positive for fall risk compared to those who screened negative, (2) reported having fallen at least once during the past year compared to those who reported no falls, and (3) reported having experienced at least one injurious fall during the past year compared to those who reported no injurious falls. (Table 1).

### Logistic regression analyses

Table 2 presents the results from logistic regression models predicting whether patients received at least one medication-related recommendation following a medication review. The adjusted odds ratios control for age, gender, average number of prescriptions filled/month, and DBI. Even after controlling for other variables in the model, the odds of receiving at least one medication-related recommendation following a medication review increased with DBI scores. For example, compared to patients with DBI scores of 0, the adjusted odds of receiving a medication-related recommendation were 18.77 times greater among those with Very High DBI scores (95% CI: 6.20, 56.81). In contrast, controlling for DBI, average number of prescriptions filled/month was not associated with the odds of receiving a medication-related recommendation.

**Table 2:** Logistic regression analyses predicting odds of pharmacist communication at least one medication recommendation to patient’s health care provider following medication review

Predictor	Unadjusted Odds Ratio (95% Wald CI)	Adjusted Odds Ratio (95% Wald CI)
Age	1.00 (0.98, 1.02)	1.0 (1.00, 1.06)
Prescription Fills	1.08* (1.03, 1.13)	0.99 (0.93, 1.05)
Female (Reference Group)	–	–
Male	1.08 (0.66, 1.77)	1.00 (0.59, 1.70)
DBI = 0 (Reference Group)	–	–
Low DBI	3.2 (1.03, 9.97)	3.38 (1.08, 10.61)
Moderate DBI	7.09 (2.49, 20.18)	7.76** (2.69, 22.41)
High DBI	8.12 (2.97, 22.19)	9.91*** (3.49, 28.15)
Very High DBI	13.29 (4.94, 35.77)	18.77*** (6.20, 56.81)

*P < 0.01 **P < 0.001 *** *P* < 0.0001

Notes. DBI scores categorized as: Reference Group: 0, Low: > 0 to < 0.20, Moderate: 0.20 to < 0.50, High: 0.50 to < 1.0, and Very High: ≥ 1.0. Adjusted odds ratios are adjusted for all of the predictor variables shown. *CI* Confidence Interval

Table 3 presents the results of logistic regression models predicting the secondary outcome variables: screening positive for fall risk, reporting at least one fall within the past year, and reporting at least one injurious fall within the past year. The adjusted odds ratios in the first set of columns indicate that, compared to patients with DBI scores of 0, the odds of screening positive for fall risk were 2.41 times greater among those with Moderate DBI scores (95% Confidence Interval, CI: 1.54, 3.78), 3.08 times greater among those with High DBI scores (95% CI: 2.02, 4.69), and 3.27 times greater among those with Very High DBI scores (95% CI: 2.07, 5.16). Further, the adjusted and unadjusted odds ratios for DBI were very similar and this same pattern of findings is evident for the other two secondary outcome variables. As was observed in relation to the odds of making at least one medication-related recommendation, after controlling for DBI, average number of prescriptions filled/month was not associated with any of the secondary outcomes.

**Table 3:** Logistic regression analyses predicting odds of screening positive for falls risk, experiencing at least one fall, and experiencing at least one injurious fall

Predictor	Screened Positive		1+ Fall		1+ Injurious Fall	
	Unadjusted Odds Ratio (95% CI)	Adjusted Odds Ratio (95% CI)	Unadjusted Odds Ratio (95% CI)	Adjusted Odds Ratio (95% CI)	Unadjusted Odds Ratio (95% CI)	Adjusted Odds Ratio (95% CI)
Age	0.99 (0.98, 1.01)	1.00 (0.99, 1.02)	0.99 (0.97, 1.00)	1.00 (0.98, 1.02)	0.98 (0.95, 1.00)	0.99 (0.97, 1.01)
Prescription Fills	1.07*** (1.04, 1.11)	1.02 (0.98, 1.06)	1.07*** (1.04, 1.10)	1.01 (0.97, 1.05)	1.06* (1.03, 1.10)	1.00 (0.95, 1.05)
Female (Reference group)	–	–	–	–	–	–
Male	0.74 (0.56, 0.97)	0.69 (0.52, 0.93)	0.88 (0.65, 1.20)	0.83 (0.60, 1.14)	0.89 (0.61, 1.31)	0.81 (0.54, 1.19)
Predictor	Screened Positive		1+ Fall		1+ Injurious Fall	
	Unadjusted Odds Ratio (95% CI)	Adjusted Odds Ratio (95% CI)	Unadjusted Odds Ratio (95% CI)	Adjusted Odds Ratio (95% CI)	Unadjusted Odds Ratio (95% CI)	Adjusted Odds Ratio (95% CI)
DBI = 0 (Reference group)	–	–	–	–	–	–
Low DBI	1.54 (1.00, 2.38)	1.55 (1.00, 2.39)	1.68 (0.94, 2.99)	1.68 (0.94, 3.0)	2.22 (1.01, 4.89)	2.24 (1.01, 4.95)
Moderate DBI	2.42*** (1.55, 3.78)	2.41*** (1.54, 3.78)	2.71** (1.55, 4.74)	2.70** (1.54, 4.75)	2.43 (1.10, 5.38)	2.46 (1.11, 5.46)
High DBI	3.10*** (2.07, 4.64)	3.08*** (2.02, 4.69)	3.01*** (1.80, 5.04)	3.00*** (1.77, 5.09)	3.18* (1.54, 6.55)	3.16* (1.51, 6.60)
Very High DBI	3.45*** (2.35, 5.08)	3.27*** (2.07, 5.16)	4.75*** (2.90, 7.76)	4.64*** (2.67, 8.07)	5.48*** (2.76, 10.89)	5.32*** (2.51, 11.27)

*P < 0.01 **P < 0.001 *** *P* < 0.0001

Note. DBI scores categorized as: Reference Group: 0, Low: > 0 to < 0.20, Moderate: 0.20 to < 0.50, High: 0.50 to < 1.0, and Very High: ≥ 1.0. Adjusted odds ratios are adjusted for all of the predictor variables shown. *CI* Confidence Interval

### Risk of falling and power

Among all participants who were screened for fall risk, 312 (29.5%) reported having fallen at least one time during the past year. The risk of reporting a fall increased monotonically from 13.2% among participants with DBI scores of 0 to 42.0% among those with DBI scores of 1.0 or greater. We performed power analyses to assess the effect of restricting participants to those with DBI scores ≥1.0. In these analyses, we assumed two groups with an equal number of participants in each group. We specified that the intervention cut the risk of falling in half (Relative Risk = 0.5) and set alpha (2-tailed) at 0.05. When the risk of falling in the control group was set at 0.295, consistent with the percentage of falls experienced in our full sample, a total of 248 participants would be needed to achieve a power of 0.80. With the risk of falling set at 0.42, consistent with the percentage of falls experienced by participants in our study who had DBI scores ≥1.0, 152 participants would be needed to achieve the same level of power.

## Discussion

Our findings extend previous research by demonstrating an association between DBI scores and the risk of *medication-related* falls. Participants were limited to patients at high risk for falls due to either polypharmacy, defined as using 4+ chronic medications, or using at least one FRID. These eligibility criteria are similar to those used in other fall prevention interventions focused on medication review and modification [18–20]. Overall, 29.5% of participants reported having fallen during the past year, compared to 42.0% of those with DBI scores ≥1.0. Further, among patients who screened positive for fall risk and received a medication review, the review resulted in a medication-related recommendation for only about 10% of those who were not taking any DBI drugs, compared to over 60% of those with DBI scores ≥1.0. Finally, although we found associations between all of the outcomes examined and the average number of prescriptions filled/month, these relationships became nonsignificant after controlling for DBI.

Our findings have implications for both research and practice. In terms of research, statistical power is affected by baseline risk [22]. In general, the rarer the outcome of interest, the more participants needed to achieve the same level of power. We demonstrated that sample size requirements in fall prevention interventions focused on medication review and modification could be reduced substantially by using the DBI to limit participants to those at greatest risk of falling. We also demonstrated that patients with low DBI scores are unlikely to benefit from medication reviews because the reviews are unlikely to result in recommendations for medication modifications. Therefore, excluding patients with low DBI scores could increase the effect size observed.

With respect to practice, both polypharmacy and many specific medications have been associated with an increased risk of falling [9, 38]. However, evidence-based guidelines to identify individuals at greatest risk for *medication-related* falls are lacking. Zia and colleages [15] found that use of 2+ FRIDs was more strongly associated with the risk of falling than polypharmacy. The Merit-based Incentive Payment System (MIPS), administered by the Center for Medicare and Medicaid Services, includes a quality measure based on the percentage of patients taking high-risk medications. Although some medications included in the MIPS have dose and days supply criteria, the measure simply assesses the percentage of patients who meet the criteria (e.g., taking more than X mg/day of a specific medication). In contrast, the DBI assesses total exposure to high risk medications by incorporating multiple pieces of information concerning the dosage regimen into a single metric. Potentially, the DBI could be used in practice to identify patients at greatest risk for *medication-related* falls and serve as the basis for referrals to pharmacists specializing in geriatric pharmacotherapy for follow-up.

This study has three primary limitations. First, DBI scores did not incorporate the use of nonprescription medications. Inclusion of these medications would likely have strengthened the relationships observed. Second, restricting study participants to people who primarily used a single pharmacy to obtain most of their medications may limit the generalizability of study findings. Third, we did not control for the presence of comorbidities and other risk factors that may have contributed to the falls experienced by study participants and these factors may have been more prevalent among individuals with high DBI scores compared to those with lower scores. However, previous research has demonstrated that a high DBI score is an independent risk factor for falls, even after controlling for comorbidities and other risk factors [26, 32, 33]. Our findings extend this previous research by demonstrating that, even in a sample selected for use of high-risk medications, the DBI can be used to identify individuals most likely to have a medication-related problem, as indicated by pharmacist recommendations for medication regimen changes, that could contribute to the risk of falling. Thus, despite these limitations, our findings suggest that the DBI is a useful tool that could be used to improve future research and practice by focusing limited resources on those individuals at greatest risk of *medication-related* falls.

## Data Availability

The datasets that support our findings are not publically available. In this project, they were used under a data use agreement with the Centers for Medicare and Medicaid Services (RSCH-2016-28662).

## Abbreviations

CI: Confidence Interval
DBI: Drug Burden Index
FRIDs: Fall-risk-increasing-drugs
IQR: Interquartile Range
IRB: Institutional Review Board
MIPS: Merit-based Incentive Payment System
NC: North Carolina
SAS PC: Statistical Analysis System for Personal Computers
SD: Standard Deviation
STEADI: Stopping Elderly Accidents, Deaths & Injuries
